# A Bayesian method for inferring quantitative information from FRET data

**DOI:** 10.1186/2046-1682-4-10

**Published:** 2011-05-09

**Authors:** Catherine A Lichten, Peter S Swain

**Affiliations:** 1 Department of Physiology, McGill University, 3655 Promenade Sir William Osler, Montreal, Quebec H3G 1Y6, Canada; 2 Centre for Systems Biology at Edinburgh, University of Edinburgh, Edinburgh EH9 3JD, UK

## Abstract

**Background:**

Understanding biological networks requires identifying their elementary protein interactions and establishing the timing and strength of those interactions. Fluorescence microscopy and Förster resonance energy transfer (FRET) have the potential to reveal such information because they allow molecular interactions to be monitored in living cells, but it is unclear how best to analyze FRET data. Existing techniques differ in assumptions, manipulations of data and the quantities they derive. To address this variation, we have developed a versatile Bayesian analysis based on clear assumptions and systematic statistics.

**Results:**

Our algorithm infers values of the FRET efficiency and dissociation constant, *K_d_*, between a pair of fluorescently tagged proteins. It gives a posterior probability distribution for these parameters, conveying more extensive information than single-value estimates can. The width and shape of the distribution reflects the reliability of the estimate and we used simulated data to determine how measurement noise, data quantity and fluorophore concentrations affect the inference. We are able to show why varying concentrations of donors and acceptors is necessary for estimating *K_d_*. We further demonstrate that the inference improves if additional knowledge is available, for example of the FRET efficiency, which could be obtained from separate fluorescence lifetime measurements.

**Conclusions:**

We present a general, systematic approach for extracting quantitative information on molecular interactions from FRET data. Our method yields both an estimate of the dissociation constant and the uncertainty associated with that estimate. The information produced by our algorithm can help design optimal experiments and is fundamental for developing mathematical models of biochemical networks.

## Background

Proteins work together continuously in the cells of all living things, generating cascades of reactions that are vital for life. To fully understand each individual protein's task requires discovering the timing, location, and strength of its interactions. To acquire this detailed information, fluorescence microscopy methods are ideal because they can provide dynamic, single-cell data at high spatial resolution [1,2]. One fluorescence tool that enables researchers to observe protein interactions in living cells is Förster resonance energy transfer (FRET). FRET data has the potential to yield biochemical constants, which are critical for modeling biological systems, but measuring protein interactions from FRET data requires careful quantitative analysis.

FRET is a physical process where a molecule in an excited energetic state (the donor) transfers energy to a nearby ground-state molecule (the acceptor). The chance that an excited donor will transfer its energy to an acceptor (known as the FRET efficiency, *E_fr_*) depends on the distance between the donor and acceptor (which must be between 1 and 10 nm for FRET to occur), their relative orientation, and the extent to which the donor's fluorescence emission peak overlaps the excitation peak of the acceptor [3]. Optimal conditions for energy transfer occur when the distance between donor and acceptor is minimal, the molecules' electric dipoles are aligned, and the spectral overlap is significant.

When FRET is used to study protein interactions in living cells, the proteins under investigation are fused to fluorescent tags (often variants of the green fluorescent protein) that act as the donors and acceptors. When the proteins interact, they bring the fluorescent tags together so that FRET may occur. FRET increases the number of photons emitted by acceptors and reduces both the number of photons emitted by donors and the donor's fluorescence lifetime. To observe these effects, the most common techniques for collecting FRET data include fluorescence lifetime imaging (FLIM) and using a fluorescence microscope or spectrofluorometer to record fluorescence intensity after exposing samples to light that mainly excites either donors or acceptors [4-6].

For FRET data to reveal information about the underlying protein interactions, complicating factors must be dealt with. One confounding issue is spectral contamination, which arises from the requirement that the donor and acceptor must have overlapping spectra for FRET to occur. Due to the overlap, excitation light intended to excite one fluorophore may also excite the other (spectral crosstalk), and, conversely, one fluorophore may emit photons in the emission range of the other (spectral bleed-through). Several FRET analysis methods address this issue by calculating a FRET index, which is the FRET signal corrected for contamination from spectral overlap and normalised by donor or acceptor concentrations [7-12]. Although they are straightforward to calculate, FRET indices often bear an indirect and nonlinear relationship to the underlying concentrations and strength of protein interactions [13,14]. Alternatively, the ratio of donors to acceptors and the apparent FRET efficiency, which is the product of the intrinsic FRET efficiency and the fraction of donors (or acceptors) in complex, can be calculated [15-19,14]. Such quantities can be measured, provided calibrations are carried out using constructs consisting of a donor linked to an acceptor [17,19], but again do not relate directly to the dissocation constant (*K_d_*) of an interaction.

Another significant challenge is that both the *K_d _*and the FRET efficiency, *E_fr_*, affect the observed signals, and so neither can be determined independently of the other based on data from a single sample. *E_fr _*has been estimated from separate acceptor photobleaching [14] and FLIM experiments [15], but these approaches have drawbacks. Acceptor photo-bleaching is slow, irreversible, often fails to bleach all acceptors, and yields the intrinsic FRET efficiency only when all donors are in complex with acceptors [4-6]. FLIM requires specialized equipment, is often slower than standard fluorescence imaging, and analysis of FLIM data is complicated by the multiexponential fluorescence decays of fluorescent proteins [20]. There is however an alternative to directly measuring *E_fr_*: Many values of *K_d _*and *E_fr _*will be consistent with data from a single cell or sample, but, by taking a set of data from samples that contain varying concentrations of donors and acceptors and analyzing it altogether, it is possible to find the unique values of *K_d _*and *E_fr _*that are consistent with the ensemble [16,21].

Given these different approaches, it is not always obvious which one should be applied in different situations and there is no consensus on the statistical analysis, with each method processing the data differently and most giving no procedure to test the reliability of any estimates. A general method for inferring the *K_d _*along with the uncertainty of that inference is necessary for the standardization of quantitative FRET measurements, the design of informative experiments, and for providing *in vivo *parameters for developing models of protein networks [22-24].

Here we propose a systematic analysis method that explicitly includes models of the photophysics and underlying chemical interactions and of measurement noise. Building on a spectral model for FRET [25], we develop a Bayesian algorithm to infer both the interaction strength (in terms of the *in vivo K_d_*) and the FRET efficiency. Applying our algorithm to simulated data, we gain insight into how both experimental design factors such as measurement noise, number of measurements, fluorophore concentrations and ratios, and prior information impact our estimate and its uncertainty.

## Results and Discussion

### Algorithm

#### Overview of the mathematical model

**Figure 1:**
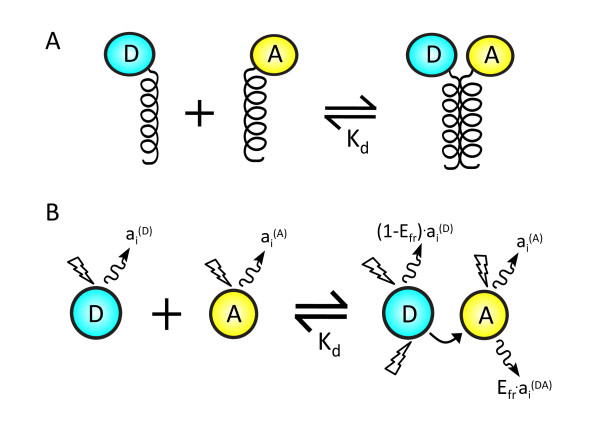
**Overview of interaction and photophysical model**. (A) In the underlying interaction, a donor-tagged protein binds an acceptor-tagged protein with dissociation constant *K_d_*. (B) During a FRET experiment, photophysical events occur such as FRET, which occurs between donors and acceptors in complex with efficiency *E_fr_*, and the excitation of and emissions from donors and acceptors, where the factor relating fluorescence observed in channel *i *to the concentration of species *S *is . We include all possible spectral overlap effects, allowing the donor and acceptor to be excited and emit photons in all spectral channels.

FRET enables the study of molecular interactions in diverse settings. To design a widely applicable analysis technique, we consider a general system containing proteins (or other molecules) that form complexes and are labelled with fluorophores that act as FRET donors and acceptors (Figure 1A). We assume that all the molecules of interest are fluorescently tagged, so instead of referring to 'donor-tagged proteins' or 'acceptor-tagged proteins', we simply refer to 'donors' and 'acceptors'.

Our model relies on a few other assumptions. First, we assume that donors and acceptors may be free or form bimolecular, donor-acceptor complexes (i.e. [*D*] + [*A*] ⇌ [*DA*]) with dissociation constant . Whether free or in complex, the fluorophores undergo fluorescence excitation and emission (Figure 1B). In complexes, the donor and acceptor are together and FRET may also occur. We assume that FRET due to random collisions between donors and acceptors occurs seldom enough to be neglected. Finally, we assume that donor-acceptor complexes all have the same FRET efficiency, which is denoted *E_fr _*and refers to the fraction of instances where exciting a donor in complex leads to excitation of the acceptor.

For our general mathematical description of the FRET system, we use a previously described spectral mixing model [25] with slightly modified matrices. This model relates concentrations of fluorescent molecules to the fluorescence observed at various excitation wavelengths and ranges of emission wavelengths (spectral channels). It can be written as a matrix equation in the following form [6]:(1)

where **C **is a vector of the concentrations of fluorophores (**C **= ([*D*], [*A*], [*DA*])*^T ^*), **I_obs _**is a vector of the fluorescence intensities observed for each spectral channel, and *M *is a matrix of the photophysical parameters that relate the concentrations of fluorophore to the observed intensities (Figure 1B).

The observed fluorescence intensities could represent data obtained from fluorescence microscopy, spectrophotometry or a flow cytometer. Microscopy images would require standard image processing steps for quantification, such as subtracting background fluorescence and defining regions of interest such as areas within the cytosol or nucleus for localized fluorescence [26].

#### Photophysics in the mathematical model

The matrix *M *contains all the relevant information about the photophysical processes we model. In addition to the expected direct excitation and emission, we include resonance energy transfer, cross-talk (unintended excitation) and bleed-through (acceptors emitting in the donors' typical emission range).

The spectral mixing framework can represent any number of spectral channels by increasing the dimensions of the matrix, but we focus on the particular case of three, which describes the most common 'three-cube' FRET experiments [12,7-9,14,18,17,21,16]. The first spectral channel, known as the donor channel, consists of an excitation wavelength that primarily excites donors and an emission filter that collects photons from the donor's emission range. The second channel, the acceptor channel, consists of an excitation wavelength that primarily excites acceptors and an emission filter that collects photons from the acceptor's emission range. The third channel, the FRET channel, combines the donor channel's excitation wavelength with the acceptor channel's emission filter, so that donors are excited preferentially and the emissions are filtered to primarily collect photons from acceptors. For three-cube FRET, the detailed form of Eq (1) is(2)

where . The parameters  each represent a combination of excitation and emission information [25]:(3)

Here the subscript *i *refers to the spectral channel (for the three-cube case, it is 1 for the donor channel, 2 for the acceptor channel, and 3 for the FRET channel) and the superscript (*S*) refers to the fluorescent species (D, the donor; A, the acceptor; and DA, the complex). The variable *I*_*i *_is the illumination intensity for the excitation wavelength of channel *i*;  is the molar extinction coefficient of species (*S*) with excitation wavelength from channel *i*; *Q*^(*S*) ^is the quantum yield of species (*S*); and  is the product of the emission of species (*S*) at the wavelengths of channel *i *and the sensitivity of the detector to the emitted photons. We describe a calibration procedure for obtaining these values in the Methods.

To illustrate Eq (2), consider the expression it yields for , the intensity in the donor channel. The intensity will be the sum of contributions from free donors, free acceptors, and donor- acceptor complexes. The contribution from free donors is  and the contribution from free acceptors is , which results from cross-talk and bleed-through. The complex, *DA*, can potentially produce contributions from donors not undergoing FRET , from acceptors  and from FRET . Adding these contributions together, we obtain the following expression for the predicted intensity in the donor channel, ,(4)

Continuing the matrix multiplication yields analogous expressions for  and .

#### Molecular interactions in the mathematical model

To include *K_d _*in our model, we model the chemical equilibrium, [*D*] + [*A*] ⇌ [*DA*]. Defining [*D*_0 _] = [*D*] + [*DA*] and [*A*_0_] = [*A*] + [*DA*], and given that , we can compute the values of [*DA*], [*D*], and [*A*] in terms of [*D*_0_], [*A*_0_], and *K_d_*.(5)

with [*D*] = [*D*_0_] - [*DA*] and [*A*] = [*A*_0_] - [*DA*].

#### Bayesian inference

Our aim is to infer the values of *K_d _*and *E_fr _*from the data. To this end, Bayesian inference is an ideal tool. Given a model, it quantifies the knowledge gained from a set of experimental observations about the parameters of interest, including the uncertainty of that knowledge [27]. That is, it allows us to update the prior probability distribution, *P *(*K_d_*, *E_fr_*), which represents what is known about the parameters before data has been acquired, to the posterior probability distribution, *P *(*K_d_*, *E_fr_*|data), which reflects both the prior information and what is learned from the data. According to Bayes' theorem,(6)

The likelihood function, *P *(data|*K_d_*, *E_fr_*), can be explicitly expressed through our photophysical model and a model of measurement noise. It quantifies the likelihood of different values for *K_d _*and *E_fr _*given the current data. We assume that the data follows a Gaussian distribution centred around the predicted intensity given by Eq (2). For three measurements, one from each of the three spectral channels, the likelihood is:(7)

The observed intensities for each channel are *I_D_*, *I_A_*, and *I_F_*. ,  and  denote the predicted intensities and *σ_D_*, *σ_A _*and *σ_F _*measure the magnitude of the measurement noise in each channel and are approximated by the variances of the data in that channel (Methods). For fitting, we use a Gaussian approximation of Eq (7) based around the values of *D*_0 _and *A*_0 _that maximize the likelihood (see section on marginalization of *D*_0 _and *A*_0 _in the Methods). Combining the likelihood and prior probability distribution allows us to calculate the posterior probability distribution and determine the most probable values for our parameters of interest.

#### Illustration of method

**Figure 2:**
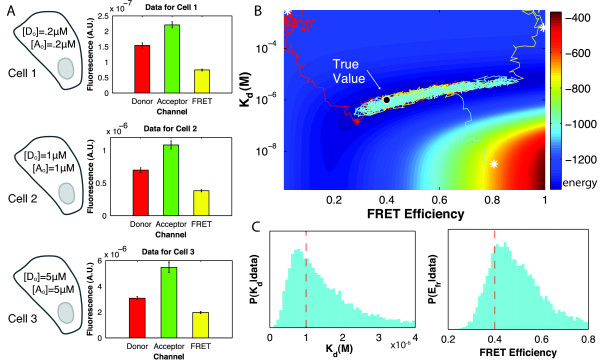
**Analysis of typical FRET data recovers true values of K_d _and E_fr_**. A typical three-cube FRET experiment is simulated from three virtual cells, each containing the indicated concentrations of donor- and acceptor- tagged proteins (A, left). The data is summarized in bar graphs with mean ± SD (A, right). Ten measurements/channel are made for each cell with 5% added measurement noise. For other parameters, see Methods. The data was analyzed in two ways to find the values of *K_d _*and *E_fr _*consistent with the data. We first calculated the energy for an array of parameter values; as the energy contour plot shows (B), the energy was minimal near the true parameter values. We also used a Monte Carlo algorithm to explore *K_d_*-*E_fr _*space, running 3 biased random walks starting from different initial positions (white *) and running for 12,000 steps. The paths of the walks (for clarity only the first 2,000 steps are shown) are superimposed on the contour plot (B) and all three converged to a region around the true value which coincides with the energy minimum. Histograms of the locations visited by all three walks, including only post-convergence steps (11,000 steps are included from each walk) act as approximate posterior distributions for *K_d _*and *E_fr _*(C). The dashed red lines indicate the true values of *K_d _*and *E_fr _*used to generate the data.

To illustrate our method, we simulated data from three cells (Figure 2A, left), although in practice, data could come instead from cellular compartments or other sub-cellular regions of interest. For simplicity, each cell contains equal concentrations of donor ([*D*_0_]) and acceptor ([*A*_0_]). In the first cell, [*D*_0_] = [*A*_0_] = 0.2 *μ*M; in the second, [*D*_0_] = [*A*_0_] = 1 *μ*M; and in the third, [*D*_0_] = [*A*_0_] = 5 *μ*M. Other parameter values are given in the Methods. We then simulated three-cube FRET measurements, generating data for the donor, acceptor, and FRET channels (Figure 2A, right). We made a series of ten measurements for each channel and each cell, to which we added Gaussian noise so that the standard deviation of the measurements was around 5% of the mean signal strength. We define *r *as the ratio of the standard deviation of the measurements to the mean signal strength; in this case, *r *≈ 0.05.

To obtain the posterior probability distribution that corresponds to this data for two parameters of interest, *K_d _*and *E_fr_*, we used two methods, the results of which should agree. First, because we are restricting our analysis to two parameters, we can visualize the distribution by computing the energy, the negative logarithm of the posterior probability, over a grid of values of *K_d _*and *E_fr_*. The resulting energy surface, shown in Figure 2B, was minimal around the true values of the parameters. If we were to extend our analysis to more than two parameters, including uncertainty in the  constants for instance, we would use a Markov chain Monte Carlo (MCMC) algorithm to sample from the parameter space. To show that the MCMC algorithm gives results consistent with the energy surface, we used the algorithm to sample the posterior probability distribution for *K_d _*and *E_fr_*. We ran the algorithm three times, starting each time at different initial values. All three walks converged on the same small region of equally probable values: they are superimposed on the energy surface in Figure 2B. Both methods therefore recover the true values used to generate the data as optimal parameter values. The approximate posterior probability distributions for *K_d _*and *E_fr _*are shown in Figure 2C. The distributions are estimated as the histograms of the three walks, excluding the burn-in period before convergence [28]. They peak near the parameter values used to simulate the data, indicating that the highly probable values for *K_d _*and *E_fr _*predicted by the methods are accurate.

#### Inferring other quantities from FRET data

To further illustrate the versatility of our method, we show that we can also use our model and Bayesian framework to estimate other parameters. Two instrument-independent parameters that have been a focus of interest are the apparent FRET efficiency, , and the ratio, [15-19,14]. The analogous apparent FRET efficiency for the acceptor, , is the product of *E_d _*and *r_da_*. We can estimate these quantities using our method, provided that a calibration has been carried out with cells expressing only donors, only acceptors, and only a donor-acceptor construct. The FRET efficiency of the construct need not be known. Supposing we have calibrated the parameters  of Eq 2 relative to , which is taken to be unity, then we can express Eq 2 just in terms of [*A*_0_] with [*D*_0_] = *r_da_*[*A*_0_] and *E_fr_*[*DA*] = *E_d _r_da_*[*A*_0_]. We can analytically maximize the posterior probability of *r_da _*and *E_d _*as a function of [*A*_0_] by setting the derivative of the probability with respect to [*A*_0_] equal to zero and solving for this optimal [*A*_0_] in terms of *r_da _*and *E_d_*. Our MCMC algorithm can be used to sample values of *r_da _*and *E_d _*that are consistent with the data.

### Testing

#### Algorithm reflects data quality

An algorithm for estimating parameter values should report not just an estimate of the most probable parameter values but also the reliability of that estimate. To evaluate this aspect of our algorithm, we measured both the error and the uncertainty of its output. We defined the error as the discrepancy between the mean of the posterior probability distribution and the true value. We calculated error as , meaning that a perfect estimate would have zero error. Because under experimental conditions we would not know the true value, we also calculated the uncertainty of the estimate as an indicator of the reliability even when true values are unknown. The uncertainty corresponds to the width of the posterior probability distributions for *K_d _*and *E_fr_*. To prevent the magnitude of the parameter of interest from skewing the uncertainty, we use the relative uncertainty, calculated as the coefficient of variation (the standard deviation divided by the mean).

**Figure 3:**
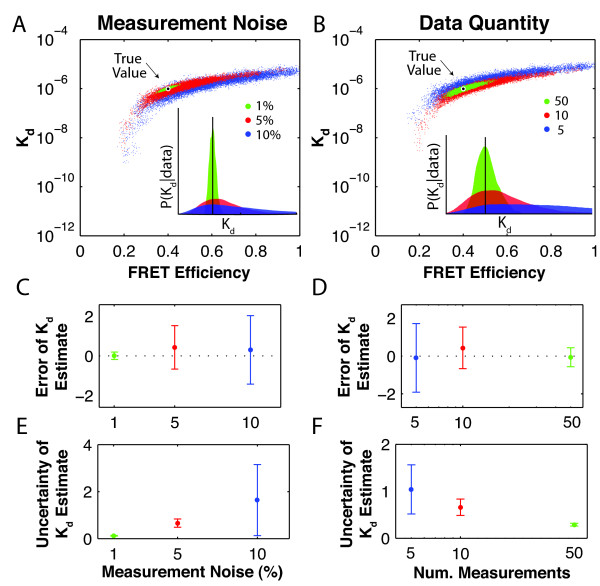
**K_d _estimates reflect data quality and quantity**. We simulated and analyzed data with varying levels of measurement noise and numbers of measurements/cell/channel. The locations visited by MCMC walks (dots) for the three noise levels (A) and the three numbers of measurements/cell/channel (B) show that the highly probable region grows as measurement noise increases and as the number of measurements decreases. The 'True Value' (black and white spot) indicates values used to generate the data. Histograms of the locations visited by the walks (insets, histograms smoothed for clarity) approximate the corresponding posterior probability distributions for *K_d_*, with true values indicated by black lines. The plots of error  vs. measurement noise (C) and error vs. amount of data (D) illustrate that, in general, accuracy decreases with increasing noise and decreasing number of measurements although the mean of the error remains centered at zero. Even when the true value is unknown, the relative uncertainty of the parameter estimate (coefficient of variation of locations visited by a walk) is measurable; it also grows with increasing noise (E) and decreasing number of measurements/cell/channel (F). In (C-F), error bars are mean ± SD. 50 data sets were analyzed for each noise level or number of measurements, and each dataset was analyzed once with a random walk running for 20,000 steps, starting once the walk converged. Except when otherwise indicated, data had 10 measurements/cell/channel and 5% added Gaussian noise. For other parameters, see Methods.

We first tested our algorithm in the presence of varying levels of measurement noise. As the examples in Figure 3A suggest, when measurement noise increased, the optimal regions in *K_d _*and *E_fr_*-space grew wider and longer and the peak of the posterior probability distribution for *K_d _*often deviated from the true value (see Figure 3A inset). In Figures 3C and 3E, the error (upper plot) and uncertainty (lower plot) for estimating *K_d _*are shown and confirm that both the error and uncertainty of the estimate increased with increasing noise. Similar results were obtained for the estimate of *E_fr _*(data not shown).

Next, we verified that gathering more data would improve the quality of the estimates of *K_d _*and *E_fr_*. Such data could be obtained by, for example, collecting several images of the same sample or dividing a region of interest into subregions. The examples in Figure 3B show that with more measurements, the optimal region became smaller and the peak of the posterior probability distribution for *K_d _*moved closer to the true value (see Figure 3B inset). Figures 3D and 3F show, as expected, that increasing the number of measurements decreases the error and uncertainty for the *K_d _*estimate. The effect of data quantity on the error and uncertainty of the *E_fr _*estimate was similar (data not shown).

Overall, our algorithm reliably reflects the quality of the data analyzed in the uncertainty it gives of its estimate and accurately infers the values of *K_d _*and *E_fr _*for 10 measurements per cell per channel with 10% measurement noise. We also note that in Figures 3C and 3D, the mean error in *K_d _*remained close to zero, indicating that neither measurement noise nor number of measurements systematically biased the location of the peak.

#### The total amount of donor, *[D_0_]*, and the total amount of acceptor, *[A_0_]*, affect inference of *K*_*d *_and *E*_*fr*_

##### *Inferring unique values of K_d _and E_fr _requires variations in *[*D*_0_]* and *[*A*_0_]

A challenge in analyzing FRET data is that both *E_fr _*and *K_d _*affect the extent of FRET that may occur, making it difficult to tease out the contribution of each parameter. In general, for data from a single pair of donor and acceptor concentrations, a weak *E_fr _*and strong *K_d _*can give rise to the same observations as a strong *E_fr _*and weak *K_d_*. However, because our algorithm can analyze data from multiple cells at once, it can overcome this challenge.

**Figure 4:**
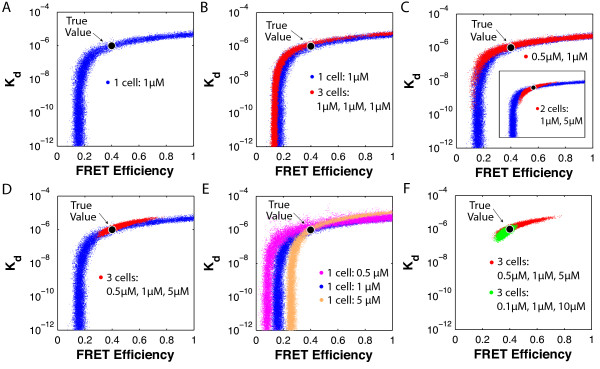
**Concentration variations determine shape of region of high probability**. Using the Monte Carlo algorithm to analyze data from a single cell containing [*D*_0_] = [*A*_0_] = 1 *μM *produced an elongated region of highly probable values (A), indicated by the collection of dots showing the locations in (*K_d_*, *E_fr_*) visited by the biased random walk. These single-cell results are shown again in (B-E) (blue dots), with the results from analyzing other data sets superimposed. Analyzing three identical cells instead of one produced a narrower but still elongated region (B). However, when analyzing data from two cells (C and C inset) and three cells (D) with different concentrations of fluorophores, the resulting regions were contracted. Analyzing cells individually showed that the elongated highly probable regions for each all intersected near the true value (E). More extreme variation in concentrations led to an even smaller optimal region (F). In (A-F), there were 10 measurements/cell/channel, 3% added Gaussian noise, and 31,000 steps are shown for each walk. For other parameter values, see Methods.

To demonstrate the problem, we analyzed a set of simulated data from a single sample with [*A*_0_] = [*D*_0_] = 1 *μM*. As Figure 4A shows, the resulting region of optimal values had an elongated shape, indicating that many equally probable solutions exist for {*K_d_*, *E_fr_*} for that dataset. To show that collecting more data from similar cells does not help, we repeated the experiment, simulating data from 3 cells with the same concentrations as the first. As Figure 4B shows, including this data made the region slightly narrower but did not change its elongated shape.

The difficulty in determining *K_d _*without knowing *E_fr _*or vice versa can be overcome by analyzing data from samples containing varying fluorophore concentrations [16,21]. To quantitatively justify how varying concentrations facilitates parameter fitting, we simulated data from three cells containing different concentrations of donors and acceptors. As Figure 4C shows, analyzing data from two different cells decreased the size of the region of equally probable values. Combining data from a cell containing [*D*_0_] = [*A*_0_] = 1 *μ*M with data from a cell containing 0.5 *μ*M eliminated high values of *K_d _*(Figure 4C); combining data from a 1 *μ*M cell and a 5 *μ*M cell eliminated low values of *K_d _*(Figure 4C, inset). As Figure 4D indicates, analyzing data from all three cells (0.5 *μ*M, 1 *μ*M, and 5 *μ*M) together eliminated higher and lower *K_d _*values, leaving a small region of equally probable values that included the true values. To show why varying fluorophore concentrations is effective, we analyzed data from each of the three cells in Figure 4D individually. The optimal regions for each cell are plotted in Figure 4E and clearly intersect at the true value. Varying the concentrations more broadly can further shrink the solution region (Figure 4F).

##### *Inference is limited if *[*D*_0_] = [*A*_0_] ≫ *K_d _or *[*D*_0_] = [*A*_0_] ≪ *K_d_*

**Figure 5:**
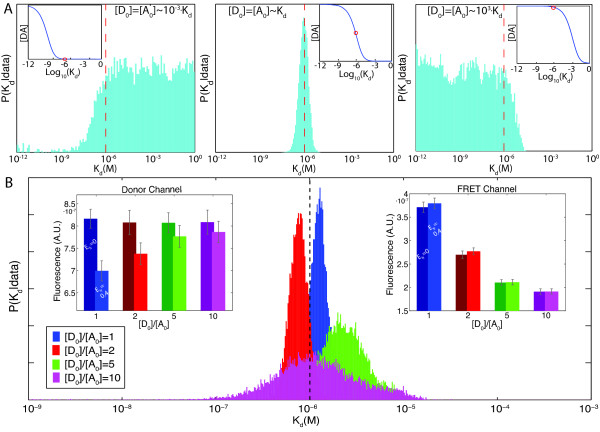
**Gaining insight into optimal experimental design**. The approximate posterior probability distributions for *K_d _*(A) have different shapes if the data analyzed was simulated from three cells containing equal concentrations of donors and acceptors which are much lower than (left), higher than (right) or about equal to *K_d _*(centre). For the data analyzed in the left panel, for instance, the cells contained the concentrations [*D*_0_] = [*A*_0_] = 0.2·10^-3 ^*μM*, [*D*_0_] = [*A*_0_] = 1·10^-3 ^*μM*, and [*D*_0_] = [*A*_0_] = 5·10^-3 ^*μM*. Insets show amount of complex formed as a function of *K_d _*for the indicated concentrations, demonstrating that where complex formation is insensitive to *K_d _*corresponds to plateaus in the posterior probability distributions for *K_d_*. In each plot (or inset), a vertical dashed line (or red circle) indicates 10^-6^*M*, the true value of *K_d_*. (A) had 36,000 steps/walk and 5% added noise. Exploring another aspect of fluorophore concentrations, increasing the ratio [*D*_0_] : [*A*_0_] increases the uncertainty in fitting *K*_*d *_(B). As the ratio was increased (by keeping [*D*_0_] constant for the three cells at 0.2·10^-6^*M*, 1.0·10^-6^*M *and 5.0 10^-6^*M *while decreasing [*A*_0_] according to the ratio), posterior probability distributions for *K_d _*broadened (true value indicated by dashed vertical line). Insets show data used for fitting (bars marked '*E_fr _*= 0.4') from the donor channel (left) and FRET channel (right) contrasted with data from the same cells simulated with *E_fr _*= 0, demonstrating that the relative contribution of FRET decreases as [*D*_0_] : [*A*_0_] increases. (B) had 50 measurements/cell/channel, 36,000 steps/walk and 3% added noise. Bars show mean ± SD. For other parameters, see Methods.

Plots of complex formation vs. *K_d _*(Figure 5A, insets) show that only *K_d _*values within a limited range significantly affect complex formation: outside this range, further changes in *K_d _*have little effect. For instance, when [*D*_0_] = [*A*_0_] ≈ *K_d_*, small changes in *K_d _*in either direction affect [*DA*] (Figure 5A centre, inset). However, when [*D*_0_] = [*A*_0_] ≪ *K_d _*(Figure 5A, left inset) or [*D*_0_] = [*A*_0_] ≫ *K_d _*(Figure 5A, right inset), only changes to *K_d _*in one direction affect the amount of [*DA*]. When a range of *K_d _*values are consistent with the data, *K_d _*cannot be fit to a single value.

By finding the posterior probability distribution of *K_d_*, our algorithm predicts when a range of *K_d _*values will be consistent with the data. First, we simulated data with equal concentrations of acceptors and donors that were very low or very high relative to *K_d_*. As shown in Figure 5A, the approximate posterior probability distributions of *K_d _*for data simulated with relatively low or high concentrations form plateaus instead of single peaks. The plateaus indicate that a range of *K_d _*values is equally probable and consistent with the data. The posterior distribution obtained for [*D*_0_] = [*A*_0_] ≪ *K_d _*reveals that *K_d _*could be any number greater than approximately 10^-6^*M*; the distribution for [*D*_0_] = [*A*_0_] ≫ *K_d _*reveals that *K_d _*< 10^-6^*M*.

This analysis illustrates how posterior probability distributions for the parameters of interest can be more informative than single values. For example, information from posterior distributions can be useful for improving experimental design. If one were to obtain plateaued distributions like the two shown in Figure 5A, one could recognize that the fluorophore concentrations were too low or high relative to *K_d _*and devise a more informative experiment.

##### The ratio [*D*_0_]:[*A*_0_] affects inference

We also used our algorithm to explore the effects of varying the ratio, [*D*_0_] : [*A*_0_], on the algorithm's ability to infer *K_d _*(Figure 5B) and how these effects arise. While other authors have reported that FRET data becomes increasingly unreliable as the ratio [*D*_0_] : [*A*_0_] deviates from unity [13], they measured how noise propagates in FRET formulas and did not directly address how the ratio impacts the measurement of interaction strength. As Figure 5B shows, the width of the approximate posterior distributions for *K_d _*broaden as the ratio increases, meaning that the uncertainty in the estimate increases. The peaks of the distributions however remain close to the true values. The same effect occurs when the ratio decreases (data not shown).

The insets in Figure 5 illustrate why the ratio affects the uncertainty, showing data from the donor and FRET channels in the presence and absence of FRET. Here, FRET contributes only a fraction of the observed signals. This small contribution is particularly evident in the FRET channel, where the change in the signal due to FRET is small compared to the measurement error: measurement error is 3% and the increase in the FRET channel from FRET is only 2.9% for [*D*_0_] : [*A*_0_] = 1 and drops to 0.7% for [*D*_0_] : [*A*_0_] = 10 (FRET is included by changing the FRET efficiency from 0 to 0.4). As the ratio increased, the relative shortage of acceptors meant that fewer complexes could form, making the contributions from FRET smaller and causing the uncertainty of the estimate to grow. In the absence of measurement noise, however, our algorithm can estimate *K_d _*even from very extreme ratios. These data also demonstrate that with our method, we can make inferences about *K_d _*even when FRET causes perceptible changes in only one channel (e.g. the donor channel here).

Knowing how spectral overlap and the ratio of donors to acceptors affect the inference of protein interaction strength helps make it possible to design informative experiments. For example, we find that in each channel, the difference generated by FRET depends on spectral overlap. In the donor channel, where FRET decreases the signal, the relative change is , meaning that the observed change depends on the difference between acceptor bleed-through, , and donor fluorescence, . Analogous changes occur in the other channels. When [*D*_0_] = [*A*_0_], the change in the donor channel is proportional to , the change in the acceptor channel to  and the change in the FRET channel to . In each channel, increasing the difference between  and  would make experiments more informative.

#### Prior information improves estimate

A further advantage of our method is its flexibility. The Bayesian framework makes it possible to incorporate additional details we know about the system as prior information, allowing us to more accurately represent the system being analyzed and potentially improve our estimate of the parameters of interest. To exploit this feature, we tested whether including additional information about the FRET efficiency would improve our inference of *E_fr _*and *K_d_*. Information about *E_fr _*could be obtained, for example, from lifetime imaging experiments [29].

**Figure 6:**
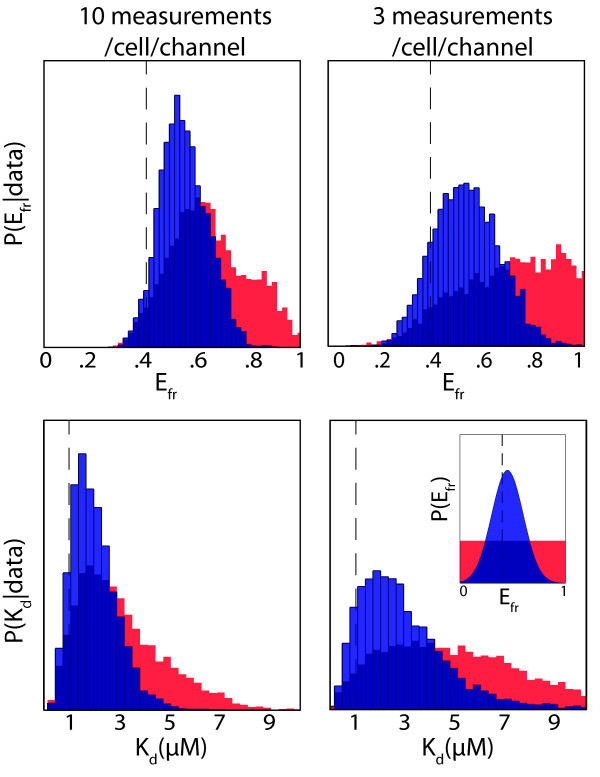
**Prior information on E_fr _improves estimate**. Approximate posterior distributions for *E_fr _*(upper panels) and *K_d _*(lower panels) obtained from using the MCMC algorithm to analyze the same datasets in the absence (red) or presence (blue) of prior knowledge about *E_fr _*show that prior knowledge helps to improve accuracy and reduce uncertainty of the estimate. True values are indicated by vertical dashed lines and the inset shows the prior distributions used (red is a uniform distribution and blue is a normal distribution centred at 0.45 with a variance of 0.15). The plots on the left show results from analyzing data where 10 measurements were made in each channel for each of 3 cells with [*A*_0_] = [*D*_0_] = [.2, 1, 5]·10^-6^*M*. The plots on the right show results from data from the same cells, but with just 3 measurements/channel. 20,000 steps were recorded for each biased biased random walk, with 5% added noise. For other parameter values, see Methods.

Figure 6 shows the approximate posterior probability distributions for *E_fr _*and *K_d _*that result from analyzing the same data in the presence and absence of prior information for *E_fr_*. We included slightly inaccurate prior information with large uncertainty, supposing that *E_fr _*was measured to be 0.45 (instead of the true value, *E_fr _*= 0.4) with an uncertainty of 0.15. Even with this limited information, the resulting histograms for both *K_d _*and *E_fr _*have taller, narrower peaks which are closer to the true values compared to the histograms produced by analyzing the same data in the absence of the prior information. As expected, this improvement was more substantial when we analyzed a set of data consisting of just 3 measurements per cell per channel (Figure 6, right column): prior information becomes more important the less relevant information there is in the data. As another form of prior information, we could also include error in the calibration measurements for the constants that relate molecular concentration to fluorescence in Eq (2). Incorporating this information would make it possible to monitor the impact of that error on the uncertainty of the final estimate of *K_d_*.

#### Summary of testing

We have shown, using typical, simulated FRET data, that our algorithm accurately recovers the parameters of interest. It responded consistently and intuitively to changes in the amount of measurement noise present in the data and the quantity of data. We also used the posterior probability distributions we obtain for the parameters to gain insight into how the magnitude and variation of the donor and acceptor concentrations affect the ability of the algorithm to infer *K_d_*. The algorithm gave informative results even when given data simulated with very high or low concentrations of donors and acceptors relative to the *K_d _*although, as expected, with this data it could not converge on a unique pair of values for *K_d _*and *E_fr _*but gave upper or lower bounds. Gaining an understanding of how experimental parameters such as measurement noise, number of measurements, spectral overlap and fluorophore concentrations affect inference allows experimenters to design optimal experiments that yield information with less uncertainty [24].

## Conclusions

Fluorescence microscopy and FRET open a window onto the cell, allowing us to observe protein interactions as the cell functions as a complete system. However, for protein interaction information from FRET data to be integrated into models and improve our understanding of biological systems, it must be reliably quantified, including the uncertainty in the estimates produced [27].

For this purpose, we have presented an algorithm for inferring the most probable values of the absolute or relative *in vivo *dissociation constant and the FRET efficiency from three-cube FRET data. Our algorithm produces estimates in terms of the posterior probability distribution for the parameters of interest. Posterior probability distributions yield more extensive information than a single value can provide, conveying the reliability of the estimates through the shape and width of the distribution and giving upper or lower bounds on parameters if the data is not more informative. Our method requires only basic three-cube FRET data but has the flexibility to use data from more spectral channels and incorporate other types of data, such as a separate measure of the FRET efficiency. We have focused on using our algorithm to fit *E_fr _*and *K_d_*, but it can also infer the apparent FRET efficiency and ratio of donors to acceptors, other quantities sometimes used as measures of FRET [15-19,14].

In the examples described here, we make a few assumptions, but these are not necessarily part of our methodology. First, we use molar extinction coefficients that must be measured separately or taken from the literature and assume that the literature measurements are valid in the cellular environment. Second, we assume Gaussian measurement noise, but our model could be straightforwardly adapted to include log-normal or other types of noise. Finally, we have not taken into consideration photo-bleaching, incomplete labelling or dark states, but our model could readily be extended to include these factors.

We have used simulated data to illustrate principles that apply to real data, showing how, in practice, it is best to infer the dissociation constant from FRET data. Three-cube FRET experiments are a well-established experimental technique [3-6]. For our method, the calibration procedure we describe in the Methods for quantifying the  constants of Eq (2) is also necessary. The procedure is similar to existing calibration methods. Measuring relative values of  and  is a standard part of FRET data collection, necessary for calibrating the extent of spectral bleed-through and cross-talk. We require calibration with a donor-acceptor construct to relate the brightness of the donor to that of the acceptor, which is necessary for obtaining the relative *K_d_*. This calibration is not always a part of standard FRET data collection, but it has been used in several studies [8,18,17,4,14,19,21]. Alternatively, measuring the absolute brightness per molecule, which is necessary for measuring the absolute *K_d_*, is more technically challenging but has been achieved [30,31].

We have demonstrated that to infer the values of *K_d _*and *E_fr_*, it is important that the FRET data analyzed come from samples containing varying concentrations of donor- and acceptor-tagged proteins. The precise amount of variation required is difficult to predict because it depends on the measurement noise present in the data, the number of measurements made, and the extent of spectral bleed-through and cross-talk. Naturally occurring variation in protein expression from cell to cell generated sufficient variability for estimating the FRET efficiency and relative dissociation constant [21], at least in transiently transfected mammalian cells.

While a number of *ad hoc *methods exist for quantifying protein interactions using FRET, our method contributes something new in that it makes plain the spectral information being used and the bio-chemical assumptions made about the system. Our model is general: it is not specific to three-cube data and could be used to analyze data with any number of spectral channels. It can be straight-forwardly adapted to other experimental situations, for instance to measure the dimerization affinity of a homodimeric protein. Our approach is a Bayesian analysis that reveals the uncertainty in estimated parameters and produces informative results for data from a wide-range of experimental set-ups. It focuses on determining what we can learn from experimental observations about the parameters of interest given clear assumptions and a systematic, statistical analysis based only on those assumptions.

## Methods

### Calibration of fluorescence constants, 

The  constants, which relate concentrations of single fluorophores to observed fluorescence intensity, can be obtained by calibration. Calibration of spectral cross-talk and bleed-through is a standard part of FRET quantification [4-6]. If the fluorophore concentrations can be determined [30,31], then the values for the three  and three  constants can be obtained from samples containing only donors or only acceptors. This calibration makes it possible to measure the absolute *K_d_*. Three-cube data from donor-only and acceptor-only samples correspond to the following equations (from Eq (2)):(8)(9)

To infer the values of the constants  and  and their uncertainty (due to measurement error), we can sample from posterior distributions for  and  given the calibration data. We will illustrate this procedure for a more complicated example below.

Determining the , which relate a complex undergoing FRET to its fluorescence in channel *i*, is more complicated because the  depend on properties of both the donor and the acceptor. However, from Eq (3), , but . Therefore, , and so  can be obtained through knowledge of the ratio of molar extinction coefficients at the excitation wavelength for channel *i*. The values of the molar extinction coefficients, * ε *^(*D*) ^and * ε*^(*A*)^, may not always be available at these wavelengths, but they can be estimated from literature values of the molar extinction coefficients (usually measured at the fluorophore's excitation peak) and the excitation spectra of the donor and acceptor [25]. Interpreting the excitation spectra as the probability of the fluorophore becoming excited and assuming that the extinction coefficient is proportional to the probability of excitation, we can use the excitation spectra to rescale the literature value of the extinction coefficient to the excitation wavelength of channel *i*. This estimate is valid provided that the molar extinction coefficients obtained from the literature are not significantly different from the molar extinction coefficients in the cellular environment of the experiment (or provided that the change in environment affects both donor and acceptor similarly).

When the relationship between brightness and concentration cannot be determined absolutely, relative values for  and  can be obtained from samples containing only donors and only acceptors, along with samples containing linked donor-acceptor constructs. The constructs consist of a donor and an acceptor fluorophore separated by a short linker of 5-10 amino acids and have been used for FRET calibration in several studies [8,18,17,4,14,19,21]. The construct's FRET efficiency, , need not be known and can be determined using the procedure we describe below.

The three-cube measurements on samples containing only donors or only acceptors would correspond to Eqs (8) and (9). Three-cube data obtained from samples containing donor-acceptor constructs would correspond to the following equations:(10)

where we have replaced  by . To infer the values of the  constants given calibration data consisting of three-cube measurements made on samples with only donors, only acceptors, and donor-acceptor complexes, we first define the general likelihood function of these experiments for a single sample:(11)

where , , and  are the *i^th ^*measurements in the donor, acceptor, and FRET channels, respectively. For a given sample, we assume that an equal number of measurements, *n*, will be made in each channel and *σ *quantifies the error of the measurements. The predicted intensities in channel *k*, , are given by Eqs (8), (9) and (10) and are a function of the constants , the concentrations of the species (donors, acceptors, or donor-acceptor complexes), and also  in the case of the donor-acceptor construct. The full likelihood for all the calibration data together would be the product of three instances of Eq (11), one specified for each three-cube experiment (donor-only, acceptor-only, or donor-acceptor complex).

We are interested in the values of the  but indifferent to the concentrations of fluorophores and complexes in the samples. For this reason and because the concentrations of fluorophores and complexes will vary for different samples, it is useful to marginalize the calibration likelihood over [*A*_0_], [*D*_0_], and [*DA*]. We can also eliminate *σ *by marginalization [28], assuming the measurement error is the same for all three spectral channels. If this assumption does not hold, one can define *σ_k _*for the measurement error in each channel and either approximate each *σ_k _*as equal to the standard deviation of the measurements in the *k^th ^*channel or include the *σ*_*k *_as parameters to be inferred in the procedure we describe below. Assuming a prior probability that only specifies positive values for the  and , marginalizing Eq (11) over the concentrations and *σ *yields the posterior probability of  and relative values of :(12)

which is valid for the three cube experiments for each sample (donor, acceptor, or construct). For the donor-only sample, ; for the acceptor-only sample, ; and for the sample with the donor-acceptor construct, the *a_i _*are replaced with . The posterior probability, including all the calibration data together, is obtained by multiplying the forms of Eq (12) for each of the three 3-cube experiments.

For a given data set, we can infer  and five of the six  constants relative to the remaining one, for instance , which is set to unity. The numerator of Eq (12) remains unaltered by such a rescaling of the , and the alteration in the denominator is cancelled by the Jacobian required for the change in variables. One can use a Markov chain Monte Carlo method to sample the  and  from *P*_calib_. Alternatively, one can use a numerical solver to find the most probable values by solving for the roots of the system of equations consisting of the derivatives of Eq (12) with respect to each of the six variables. Using this relative calibration procedure, the final *K_d _*values obtained would relate to the true *K_d _*by the scaling factor, for instance .

### Data simulation

We designed our simulated data to mimic the key features of experimental data, which could come from various sources, such as fluorescence reader measurements of solutions of purified proteins or images of cells from fluorescence microscopy that have been processed and quantified. To simulate data, we wrote a function in Matlab (The Mathworks, Natick, MA) that takes as input *E_fr_*, *K_d_*, **A_0_**, **D_0_**, *n*, , and *r *where **A_0_**, **D_0 _**are vectors of length *m *(representing *m *cells or regions within cells), *n *is the number of measurements made on each cell or vesicle, and *r *is the strength of measurement noise. The function outputs a set of simulated three-cube FRET data from *m *samples with *n *measurements per sample.

For each pair of concentrations, [*A*_0_] and [*D*_0_], we calculate [*D*], [*A*], and [*DA*] using Eq (5). Next, we calculate the simulated experimental intensities *I_D_*, *I_A_*, and *I_F _*using Eq (1). Finally, we simulate measurement noise by adding Gaussian random numbers to the data. The variable *r *is a scalar between 0 and 1 that refers to the strength of the measurement noise relative to the mean of the observed signal. Noise with *r *= 10% would have Gaussian measurement noise with standard deviation that is 10% of the mean signal observed in each channel.

In our examples, we use *E_fr _*= 0.4 and *K_d _*= 1 *μ*M. Unless otherwise indicated, we simulate ten measurements per cell per channel and use the following set of  values, which were chosen to represent a donor-acceptor pair with considerable spectral cross-talk and bleed-through:

The constants corresponding to the donor, acceptor, and complex fluorescing in their respective channels  are assigned the largest values. As donors and acceptors are often not equally bright, we set . In the FRET channel, the constants corresponding to bleed-through from donors  and cross-talk from acceptors  have been set to approximately 20% of the constants for donor and acceptor in their respective channels  because spectral overlap contributes significantly in the FRET channel. In the acceptor channel, smaller values are assigned to  and  to describe the donor undergoing cross-talk and, subsequently, either bleed-through () or FRET () in the acceptor channel. It is unlikely that acceptors would emit photons detectable in the donor channel, so the constants corresponding to that process,  and , are very small, but they are non-zero to show that any measurable spectral contamination can be included.

### Incorporating prior information

The Bayesian framework makes it possible to incorporate prior information about any of the parameters, including uncertainty in . As an example, we include prior knowledge about *E_fr _*(see the Results). Such data could be collected in separate experiments under slightly different experimental conditions and in the presence of measurement noise. We define the prior probability of *E_fr _*by a Gaussian distribution with mean at the measured value  and variance *σ_E_*, which reflects the confidence in the measurement:(13)

### Markov chain Monte Carlo (MCMC) estimation

To sample *K_d _*and *E_fr _*from the posterior probability distribution, we use a biased random walk. Although a simpler approach would be sufficient to explore two dimensions, we use this method because it will allow us to efficiently extend our search to sample other, additional parameters we may wish to infer, such as the values of .

We use the Metropolis-Hastings algorithm [32,33]. It begins at a random location in parameter space and takes random steps, moving in up to 3 dimensions at a time with proposed steps drawn from a distribution that is symmetric about the current location. Proposals that increase the posterior probability are always accepted; those that decrease it are accepted with probability , where *P *(*x^j^*) is the posterior probability of the proposed step and *P *(*x*^*j*-1^) is the posterior probability of the current location. The walk is run for a sufficiently long time (about 10,000 steps) to generate independent samples from the posterior distribution and the step size chosen to maintain an acceptance rate of 40 - 60% [28].

Once the walk has converged with the energy fluctuating around a minimum value, we record the steps taken and use the histogram of these sampled values as our estimate of the posterior probability distribution. From this estimated distribution, we obtain the mean and standard deviation of each parameter being inferred. The algorithm is summarized below.

### Marginalization of *D*_0 _and *A*_0_

We have defined the likelihood in Eq 7 and assume that the measurement noise in each channel is independent. Because we have a Gaussian model, the values of the measurement noise parameters, *σ_D_*, *σ_A _*and *σ_F _*, that maximise the posterior probability are approximately equivalent to the observed variances of the data from the donor, acceptor, and FRET channels, respectively [28]. However, it would also be possible to fit ,  and  directly, along with *K_d _*and *E_fr_*.

Although *K_d_*, *E_fr_*, *D*_0 _and *A*_0 _are important parameters, we cannot measure them directly. For our purposes, we are interested in the values of *K_d _*and perhaps *E_fr_*, but not the values of *D*_0 _and *A*_0_. Rather than fit *D*_0 _and *A*_0_, we marginalize or integrate them out:(14)

To indicate that we have no knowledge about the values of *D*_0 _and *A*_0_, we set the prior, *P *(*D*_0_, *A*_0_), to a constant for positive *D*_0 _and *A*_0 _(and 0 otherwise).

As a result,(15)

Because this expression is difficult to integrate analytically, we consider the 'energy', *E *= - log(*P*(data|*K_d_*, *E_fr_*, *D*_0_, A_0_)), and approximate *E *with a two-dimensional Taylor expansion about , where  minimizes *E *(and thus maximizes the likelihood). Ignoring terms above second order,(16)

where ∇∇*E *is the Hessian, or matrix of second-derivatives of *E *with respect to *D*_0 _and *A*_0_.

Therefore,(17)

This approximation of the likelihood results in a Gaussian integrand, which we can then integrate analytically [34]. Note that we do not use the limits of integration (0, ∞) which would be appropriate for non-negative concentration values. We instead use the limits (- ∞, ∞) to make the integral simple. It is a valid approximation provided that  and  are sufficiently large and it consistently yields appropriate results in practice.

In summary,(18)

where |*H*| is the determinant of the Hessian, . Although *K_d _*and *E_fr _*do not appear explicitly in the final form of Eq (18), both  and *H *depend on *K_d _*and *E_fr_*.

## Algorithm summary

Our algorithm for sampling from the posterior probability distribution of (*K_d_*, *E_fr_*) with a prior probability distribution for *E_fr _*consists of the following steps:

1. Perform calibration to obtain values (absolute or relative) for the  constants.

2. Define prior probability distributions for parameters to be inferred based on initial information that is known, if any.

3. Run Markov chain Monte Carlo algorithm:

Choose initial step  and compute the posterior probability, , as outlined in (b) below.

For j = 2 to n,

(a) Choose proposal step .

(b) Compute the posterior probability, , for that step:

i. Find , the values of {*D*_0_, *A*_0_} that minimise the energy of the posterior probability using nonlinear optimisation (the Nelder-Mead simplex algorithm implemented in Matlab's fminsearch function (The Mathworks, Natick, MA)).

ii. Compute  using analytical expressions for the second derivatives of *E *computed in Mathematica (Wolfram Research, Champaign, IL) and exported to Matlab.

iii. Using Eq (18), compute the likelihood, .

iv. Using Eq (13), compute the prior, .

v. Compute the posterior probability distribution, *P_j_*, which is the likelihood times the prior probability.

(c) Check whether to accept the move to 

• If *P_j _*>*P*_*j*-1_, accept.

• Otherwise, accept with probability .

Steps (a)-(c) are repeated until *j *= *n*. After an initial burn-in period where the energy reaches a minimum and the walk achieves a stationary distribution, record samples of *K_d _*and *E_fr_*.

4. Repeat step 3, varying initial (*K_d_*, *E_fr_*). To ensure convergence, the stationary distributions of all walks should overlap [28].

## Availability

We have made our data simulation and analysis software available at http://swainlab.bio.ed.ac.uk/software/FRET.

## Authors' contributions

PSS conceived of the study. CL and PSS developed the methodology. CL implemented and tested the algorithm. Both authors wrote the paper and read and approved the final manuscript.
